# Influence of pre-transplant minimal residual disease on prognosis after Allo-SCT for patients with acute lymphoblastic leukemia: systematic review and meta-analysis

**DOI:** 10.1186/s12885-018-4670-5

**Published:** 2018-07-23

**Authors:** Zhenglei Shen, Xuezhong Gu, Wenwen Mao, Liefen Yin, Ling Yang, Zhe Zhang, Kunmei Liu, Lilan Wang, Yunchao Huang

**Affiliations:** 1 Department of Hematology, The Third Affiliated Hospital of Kunming Medical University, Kunming, China; 2 Department of Hematology, The First People Hospital in Yunnan Province, Kunming, China; 3 Department of Geriatrics, The Second Hospital of Kunming, Kunming, China; 4 Department of Hematology, The Second Affiliated Hospital of Kunming Medical University, Kunming, 650031 China; 5 Department of Chest Surgery, The Third Affiliated Hospital of Kunming Medical University, Kunming, China

**Keywords:** Acute lymphoblastic leukemia, Allogeneic stem cell transplantation, Minimal residual disease

## Abstract

**Background:**

This meta-analysis was performed to explore the impact of minimal residual disease (MRD) prior to transplantation on the prognosis for patients with acute lymphoblastic leukemia (ALL).

**Methods:**

A systematic search of PubMed, Embase, and the Cochrane Library was conducted for relevant studies from database inception to March 2016. A total of 21 studies were included.

**Results:**

Patients with positive MRD prior to allogeneic stem cell transplantation (allo-SCT) had a significantly higher rate of relapse compared with those with negative MRD (HR = 3.26; *P* <  0.05). Pre-transplantation positive MRD was a significant negative predictor of relapse-free survival (RFS) (HR = 2.53; *P* <  0.05), event-free survival (EFS) (HR = 4.77; *P* < 0.05), and overall survival (OS) (HR = 1.98; *P* < 0.05). However, positive MRD prior to transplantation was not associated with a higher rate of nonrelapse mortality.

**Conclusions:**

Positive MRD before allo-SCT was a predictor of poor prognosis after transplantation in ALL.

**Trial registration:**

Not applicable.

**Electronic supplementary material:**

The online version of this article (10.1186/s12885-018-4670-5) contains supplementary material, which is available to authorized users.

## Background

Acute lymphoblastic leukemia (ALL) is a hematologic malignancy of bone marrow featured by the overproduction of immature lymphoblasts [1]. It represents 75–80% of childhood acute leukemias and 20% of all leukemias in adults, with approximately 6000 cases diagnosed every year in the United States [1, 2]. Despite evolving treatment protocols, the relapse rate is approximately 15–20% in ALL, and the cure rate is much lower after relapse [3]. These relapses are due to the persistence of residual malignant cells, namely minimal residual disease (MRD), that cannot be detected by the morphological examination of the bone marrow [4]. Great efforts have been made to standardize MRD quantification using real-time polymerase chain reaction (PCR) of immunoglobulin and T-cell receptor (TCR) gene rearrangements, real-time PCR-based detection of fusion gene transcripts [e.g., breakpoint cluster region-Abelson (BCR-ABL)] or breakpoints, and flow cytometric immunophenotyping [5, 6]. MRD allows a more precise assessment of treatment efficacy and reduction of leukemic burden [7]. It has important prognostic and therapeutic implications for adults and children with ALL [8, 9]. The UKALL 2003 trial suggested that MRD risk stratification was helpful in adjusting the treatment intensity [10].

Allogeneic stem cell transplantation (allo-SCT) is the preferred treatment for adults with relapsed disease and children with high-risk relapses [11, 12]. The SCT mortality due to relapse is 30–40% in adults and children. The treatment-related mortality is also 30–40% in adults but lowers in children [13, 14]. A body of evidence indicated a direct correlation of the likelihood of relapse after transplant with the MRD status before transplantation [15, 16]. However, this significant association was not observed in some studies [17–20]. Also, patients’ age, detection methods, and adjustment of clinical covariates largely varied among different studies [16]. Additionally, the impact of MRD on overall survival (OS) and nonrelapse mortality (NRM) remained uncertain. Therefore, this systematic review and meta-analysis was conducted to explore the impact of MRD prior to transplantation on the prognosis for patients with ALL.

## Methods

### Search strategy and inclusion criteria

The meta-analysis was performed according to the Preferred Reporting Items for Systematic Reviews and Meta-Analysis statement [21]. Studies in PubMed, Embase, and the Cochrane Library were searched from the database inception to March 2016, using the following text and/or medical subject heading terms: (1) “acute lymphoblastic leukemia” or “acute lymphoblastic leukaemia”; (2) “minimal residual disease”; (3) “relapse” or “relapse-free survival” or “leukemia-free survival” or “leukaemia-free survival” or “disease-free survival” or mortality; and (4) transplantation. The search was restricted to publications in the English language. The references of included studies were screened for potentially missing records. This systematic review with meta-analysis was not registered in a trial registry.

Studies considered for inclusion were as follows: (1) reported the detection of bone marrow MRD prior to allo-SCT in patients with ALL; (2) had no limitation in terms of the age of included patients; (3) cohort study, prospective or retrospective; (4) published in English; and (5) presented data on the main outcomes of relapse, relapse-free survival (RFS), event-free survival (EFS), and/or NRM. Disease-free survival (DFS) and leukemia-free survival (LFS) were interpreted as synonymous with RFS.

### Study selection and quality assessment

Two independent reviewers (ZLS and XZG) screened the citations for inclusion based on titles and abstracts. Multiple studies involving the same cohort of patients (or duplicate patient populations) were identified and combined. Only the most recent or comprehensive study was selected to avoid double-counting. The Newcastle–Ottawa Scale (NOS) for nonrandomized studies was used to assess the quality of included studies. The items included patient selection (4 points), comparability of cohorts (maximum 2 points), and outcome assessment (maximum 3 points), with a total of 9 points [22]. The data extraction and quality assessment were conducted independently by two authors (LFY and LY). The information was examined and adjudicated independently by an additional author (WWM) referring to the original studies.

### Statistical analysis

Time-to-event data were most appropriately analyzed using the hazard ratios (HRs). Thus, the HR and its 95% confidence interval CI) were used as summary effect estimates for these outcomes. Adjusted HRs were directly extracted from the results of multivariate analysis using the Cox regression model. When missing data regarding adjusted HR were encountered, it was indirectly estimated using the Kaplan–Meier (KM) curves guided by the method of Tierney et al [23]. The random-effects model was used for meta-analysis. The heterogeneity between the included studies was assessed using the Cochran Q test and the *I*^2^ statistics. A *P* value less than 0.1 or *I*^2^ values >50% was regarded as heterogeneity [24]. Subgroup analyses were performed based on the following clinical variables: study design (retrospective or prospective), region (USA, Europe, or Asia), population (adults or children), MRD assay modality [PCR or flow cytometry (FC)], and source of effect estimates (adjusted by multivariate analysis or unadjusted from KM curves). A sensitivity analysis was performed by excluding the studies one by one. Publication bias was visually explored using funnel plots and statistically assessed using Egger’s and Begg’s tests. All data were synthesized using the STATA 12.0 software and “metan” package (StataCorp LP, TX, USA). Two-tailed *P* ≤ 0.05 was considered significant for all statistical analyses.

## Results

### Literature search

**Fig. 1:**
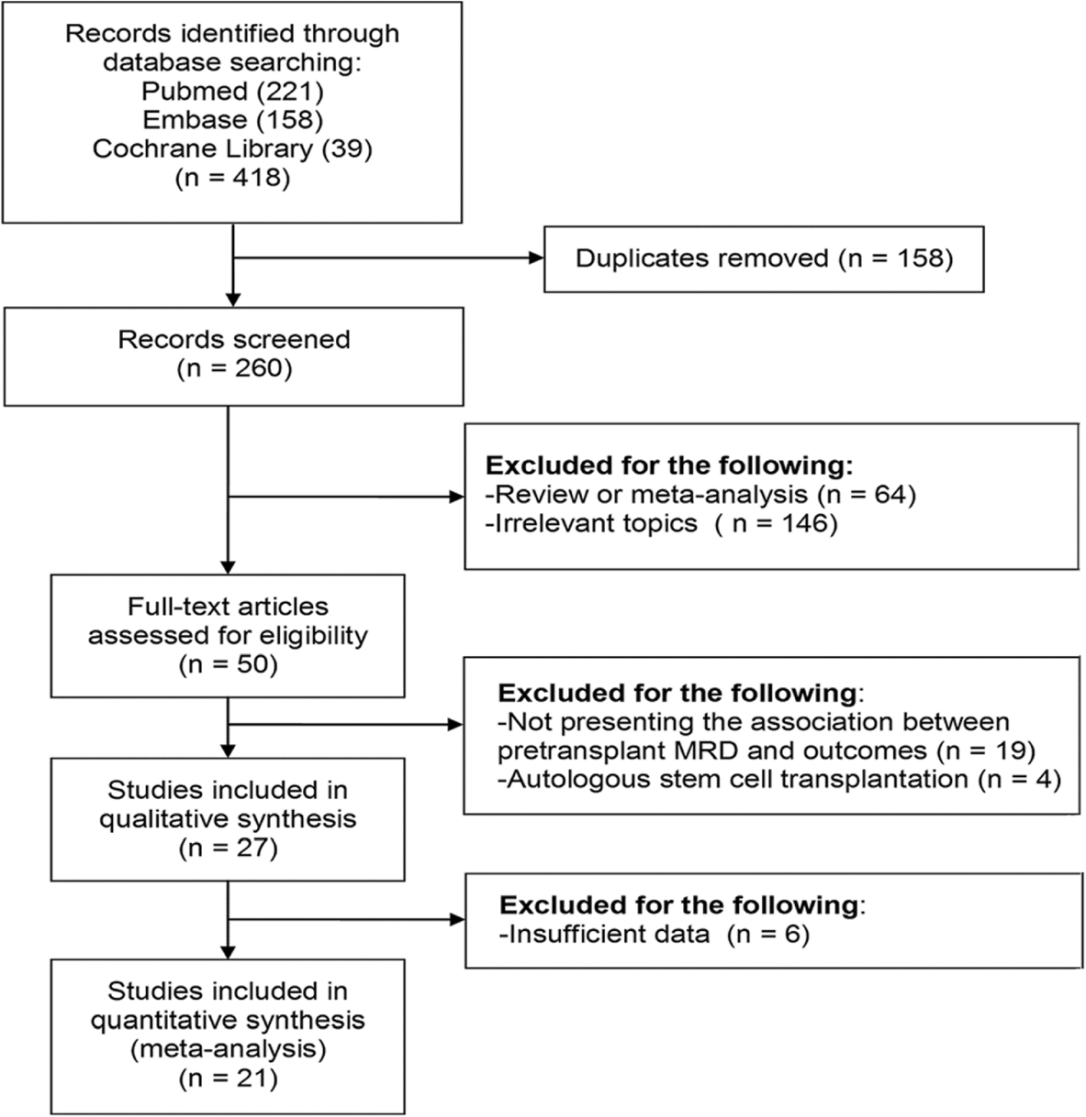
Study selection process

A total of 418 studies were identified, including 221 from PubMed, 158 from Embase, and 39 from the Cochrane Library. The 158 duplicate studies were discarded. Also, 64 reviews or meta-analyses and 146 studies on irrelevant topics were removed. Fifty full-text studies were assessed for eligibility. Moreover, 4 studies of autologous stem cell transplantation and 19 studies that did not present the association between pre-transplant MRD and outcomes were discarded. Twenty-seven studies were included in qualitative synthesis. As 6 studies had insufficient data, 21 studies were finally pooled into the meta-analysis. The flow diagram of study selection is shown in Fig. 1.

### Study characteristics and quality appraisal

The characteristics of 21 included studies are shown in Table 1. These articles were published between 1998 and 2016, including 9 retrospective studies and 12 prospective studies. The sample size ranged from 29 to 522. Seven studies enrolled mainly adult patients, 11 studies included mainly pediatric patients, and 3 studies comprised a mixture of adults and children. Six studies used FC and 15 studies employed PCR to detect MRD. The quality assessment of included studies is shown in Additional file 1: Table S1. The NOS score of included studies ranged from 5 to 9, and the important items included a representative of MRD (+) patients, comparability, and adequate follow-up duration. Seven studies did not enroll representative patients with ALL, including two studies of relapsed ALL, [25, 26] two studies of Philadelphia-positive ALL, [19, 27] one study of Philadelphia-negative ALL, [28] and two studies of high-risk ALL [18, 29]. For the item of comparability, the score was deducted when the study did not present sufficient adjusted effect estimates [14, 18, 28–33]. Only seven studies with 5-year outcomes in the follow-up were considered as the adequate follow-up [14, 18, 34–38].

**Table 1:** Characteristics of included studies

Author (year)	Design	No. of patients	Age (year)	Detection method	Endpoint	Source of HR	Adjusted covariates	Follow-up
Knechtli et al. (1998)	Retrospective	64	1.3–17	PCR IG/TCR	2-year EFS	Multivariate analysis	Philadelphia chromosome positive, pre-BMT relapse	Median: 35 months
Bader et al. (2002)	Retrospective	41	1.5–17.8	PCR IG/TCR	5-year EFS	KM curve	NA	Median: 5.75 years
Sramkova et al. (2007)	Prospective	36	1.1–19	PCR IG/TCR	4-year EFS	KM curve	NA	Median: 26 months
Spinelli et al. (2007)	Prospective	37	18–63	PCR IG/TCR, or PCR fusion transcript	3-year CIR, 3-year OS	KM curve	NA	Median: 23 months
Bader et al. (2009)	Prospective	91	3–22.6	PCR IG/TCR	4-year CIR; 4-year EFS	Multivariate analysis	Sex, age at relapse, remission status, time point of relapse, immunophenotype, site of relapse, stem-cell donor, T-cell depletion, time to transplantation, GVHD, MRD load before stem-cell transplantation	Median: 3.4 years
Elorza et al. (2010)	Prospective	31	0.8–12	FC	2-year EFS	KM curve	NA	Median: 9 months
Lankester et al. (2010)	Prospective	48	Mean: 8	PCR IG/TCR	5-year CIR, 5-year EFS	KM curve	NA	Median 61.5 months
Doney et al. (2011)	Retrospective	161	18–62	FC	5-year CIR, 5-year RFS	Multivariate analysis	Disease status, year of transplantation, CMV serology mismatch	NA
Bachanova et al. (2012)	Prospective	86	1–63	FC	2-year CIR, 3-year DFS, 3-year OS	Multivariate analysis	Disease status, ALL subtype, sex, time from diagnosis to transplantation, CMV status	Median 3.9 years
Ruggeri et al. (2012)	Retrospective	170	< 1–17	PCR IG/TCR, or fusion transcripts	4-year CIR; 4-year LFS, 4-year OS, 4-year NRM	Multivariate analysis	Age, median year of transplant, cytogenetic risk group, TBI-based conditioning regimen, number of HLA disparities	Median: 48 months
Mizuta et al. (2012)	Prospective	100	15–64	PCR BCR-ABL	3-year CIR, 3-year OS, 3-year DFS, 3-year NRM	Multivariate analysis	Age, donor status, chromosome abnormality, stem-cell source, performance status, BCR-ABL subtype, WBC, CD20	Median: 31 months
Sanchez-Garcia et al. (2013)	Retrospective	102	Mixed	FC	5-year EFS, 5-year OS, 5-year RFS	Multivariate analysis	Age, disease status, stem-cell source, time from onset to HSCT, GVHD	Median: 60.8 months
Balduzzi et al. (2014)	Prospective	82	< 1–20	PCR IG/TCR	5-year CIR, 5-year EFS	Multivariate analysis	Disease status, donor type, HLA compatibility, GVHD	Median 4.9 years
Gandemer et al. (2014)	Prospective	122	≤18	PCR IG/TCR	5-year CIR, DFS, OS	Multivariate analysis	Sex, antithymocyte globulins, CNS location, risk stratification	Median: 34.8 months
Tucunduva et al. (2014)	Retrospective	98	18–66	PCR or FC BCR-ABL	3-year CIR, 3-year LFS	Multivariate analysis	Age, sex, cytomegalovirus, disease status, transplantation method, conditioning, antithymocyte globulin, use of TKI, graft	Median: 36 months
Logan et al. (2014)	Retrospective	29	16–67	PCR	DFS	KM curve	NA	Minimum: 3 years
Zhou et al. (2014)	Retrospective	149	18–70	FC	2-year OS, 2-year PFS	Multivariate analysis	Age, disease status, allotype, cell type	NA
Bar et al. (2014)	Retrospective	160	0.6–62	FC	3-year CIR, 3-year OS	Univariate analysis	None	Median: 40.6 months
Bader et al. (2015)	Prospective	113	0–18	PCR IG/TCR	3-year CIR, 3-year EFS, 3-year NRM	Multivariate analysis	Disease status, immunophenotype, time of relapse, T-cell depletion	Range: 3.4–6.5 years
Sutton et al. (2015)	Prospective	81	< 18	PCR IG/TCR	5-year CIR, 5-year LFS, 5-year OS	Multivariate analysis	Sex, age, T-ALL, BCR-ABL1, hyperdiploidy > 50 or ETV6-RUNX1, BCP-other, IKZF1 mutation status, CR > 1, MSD, cord blood donor, mitoxantrone chemotherapy, TBI CY TT conditioning, in vitro T-cell depletion, ATG, GVHD	Median: 4.8 years
Dh’edin et al. (2016)	Prospective	522	15–55	PCR IG/TCR	3-year RFS, 3-year OS	KM curve	NA	Median: 3.5 years

*ALL* Acute lymphoblastic leukemia, *BMT* Bone marrow transplantation, *CIR* Cumulative incidence of relapse, *CNS* Central nervous system, *DFS* Disease-free survival, *EFS* Event-free survival, *FC* Flow cytometry, *GVHD* Graft-versus-host disease, *HR* hazard ratio, *IG* Immunoglobulin genes, *MRD* Minimal residual disease, *MSD* Matched sibling donor, *NRM* non-relapse mortality, *OS* Overall survival, *RFS* Relapse-free survival, *TBI* total body irradiation, *TCR* T-cell receptor genes, *TKI* Tyrosine-kinase inhibitor

### Relapse

**Fig. 2:**
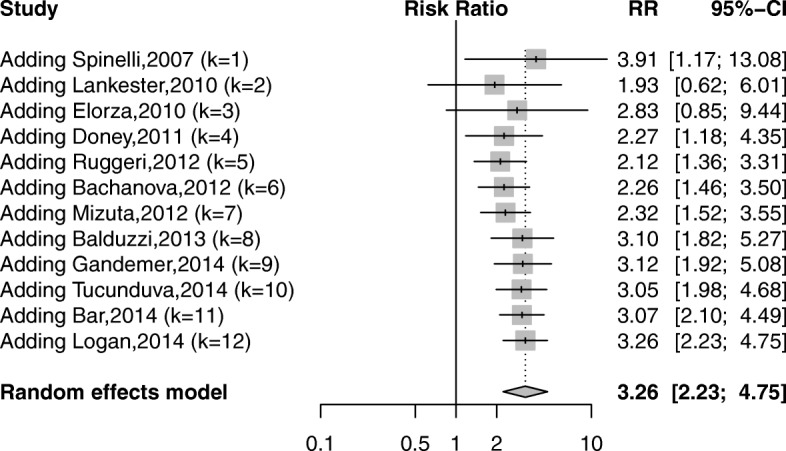
Forest plot showing the association between pre-transplant MRD and relapse after allo-SCT

Twelve studies investigated the association between pre-transplantation MRD and cumulative incidence of relapse [18, 19, 27, 29, 31–34, 36, 37, 39, 40]. HRs from five studies were derived from the KM curves, [18, 29, 31–33], and HRs from seven studies were obtained from the multivariate analysis [19, 27, 34, 36, 37, 39, 40]. Patients with positive MRD prior to allo-SCT had a significantly higher rate of relapse compared with those with negative MRD (HR = 3.26; 95% CI 2.23–4.75, *P* < 0.05) (Fig. 2). Moderate heterogeneity was revealed (*I*^2^ = 46%; *P* < 0.05). Subgroup analyses were conducted according to the following variables: population, design, region, detection method, and adjustment of HR. The pooled results remained statistically significant for all stratified analyses (except for the single Asian study), suggesting the robustness of the relationship. The heterogeneity was low or nonsignificant for the subgroups of adult patients, retrospective studies, United States of America (USA), FC, and adjusted HR, indicating that these factors might explain the observed heterogeneity (Table 2). A sensitivity analysis was further conducted. After each study was sequentially excluded from the pooled analysis, the conclusion was not affected by the exclusion of any specific study.

**Table 2:** Subgroup analyses for the outcome of relapse

Subgroups	No. of studies	HR (95% CI)	*I*^2^ (*P* value)
Population			
Children	5	3.30 (1.48–7.36)	73.1% (0.01)
Adult	4	2.90 (1.85–4.57)	8.5% (0.35)
Design			
Prospective	7	4.21 (1.93–9.16)	62.2% (0.01)
Retrospective	5	2.73 (1.96–3.79)	7.1% (0.37)
Region			
Europe	7	3.21 (1.80–5.71)	61.3% (0.02)
USA	4	3.27 (2.00–5.36)	24.6% (0.26)
Asia	1	7.34 (0.54–99.59)	–
Detection method			
PCR	7	3.82 (1.94–7.53)	62.0% (0.02)
FC	4	3.10 (1.92–5.02)	17.0% (0.31)
Adjustment			
Adjusted	7	3.24 (2.10–5.00)	32.8% (0.18)
Crude	5	3.43 (1.59–7.38)	64.6% (0.02)
Competing risk framework			
Yes	10	2.96 (2.22–3.96)	52.9%(0.02)
No	2	2.40(1.45–3.98)	0.0 (0.39)

### RFS

**Fig. 3:**
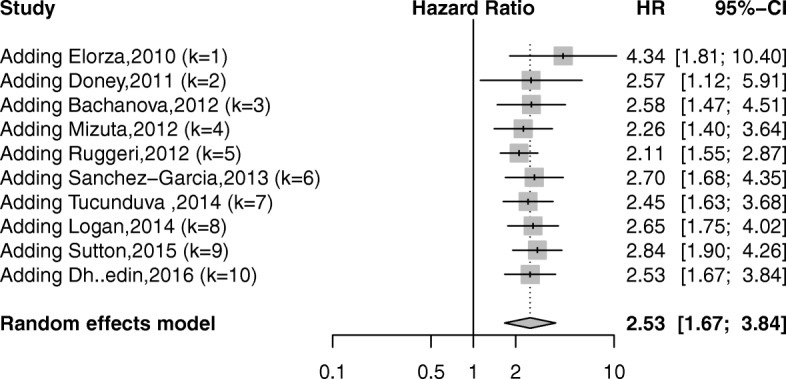
Forest plot showing the association between pre-transplant MRD and relapse-free survival after allo-SCT

Data on RFS were directly obtained from seven studies, [19, 27, 34, 35, 38–40] or indirectly calculated from three studies [28, 31, 33]. Pre-transplantation positive MRD was a significant negative predictor of RFS (HR = 2.53; 95% CI 1.67–3.84; *P* < 0.05) (Fig. 3). Statistically significant heterogeneity was revealed (*I*^2^ = 74.1%; *P* < 0.05).

Subgroup analyses were performed, and the results are shown in Table 3. Notably, the pooled results in the subgroups of Asian population and unadjusted HRs were nonsignificant. The heterogeneity remained moderate to high among all subgroups, indicating that none of the stratifying variables could explain the high heterogeneity. In a sensitivity analysis, the conclusion was not affected by the exclusion of any specific study after each study was sequentially excluded from the pooled analysis.

**Table 3:** Subgroup analyses for the outcome of relapse-free survival

Subgroups	No. of studies	HR (95% CI)	*I*^2^ (*P* value)
Population			
Children	3	3.22 (1.68–6.18)	57.6% (0.10)
Adult	4	1.69 (1.02–2.80)	69.5% (0.02)
Design			
Prospective	5	2.37 (1.12–5.01)	77.7% (< 0.01)
Retrospective	5	2.71 (1.58–4.64)	73.5% (< 0.01)
Region			
Europe	5	2.38 (1.26–4.50)	83.5% (< 0.01)
USA	3	2.87 (1.35–6.10)	56.7% (0.10)
Asia	2	2.61 (0.66–10.40)	75.6% (0.04)
Detection method			
PCR	5	2.09 (1.07–4.08)	75.3% (< 0.01)
FC	4	3.58 (1.73–7.40)	73.8% (< 0.01)
Adjustment			
Adjusted	7	2.50 (1.64–3.82)	64.4% (0.01)
Crude	3	2.93 (0.81–10.64)	86.3% (< 0.01)

### EFS

**Fig. 4:**
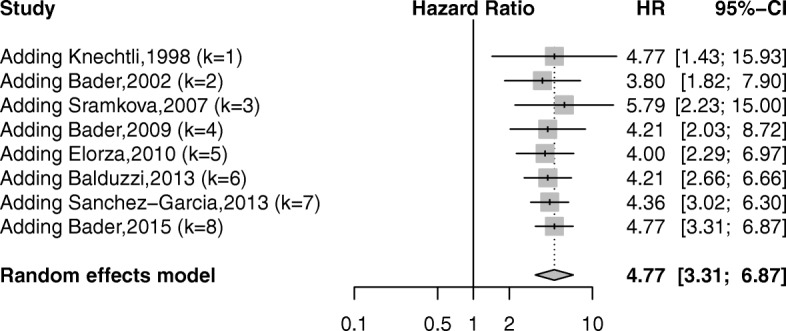
Forest plot showing the association between pre-transplant MRD and event-free survival after allo-SCT

Eight studies explored the data on EFS, with four on adjusted HRs [25, 26, 35, 41] and four on unadjusted HRs [14, 30, 31, 36]. All studies were conducted in Europe. The pooled analysis revealed a significant correlation of positive MRD before allo-SCT with worse EFS (HR = 4.77; 95% CI 3.31–6.87; *P* < 0.05) (Fig. 4). No significantly low heterogeneity was shown (*I*^2^ = 30.5%; *P* > 0.1).

In the subgroup analyses, the pooled results remained significant in all subgroups (Table 4). No heterogeneity was shown for the subgroups of pediatric patients, retrospective studies, and FC. In a sensitivity analysis, the conclusion did not change substantially by removing any single study after excluding the included studies one by one.

**Table 4:** Subgroup analyses for the outcome of event-free survival

Subgroups	No. of studies	HR (95% CI)	*I*^2^ (*P* value)
Population			
Children	6	5.62 (3.75–8.42)	3.2% (0.40)
Mixed adult and children	2	3.60 (1.72–7.51)	65.2% (0.09)
Design			
Prospective	5	5.33 (2.88–9.86)	57.6% (0.05)
Retrospective	3	4.59 (2.89–7.27)	0 (0.73)
Detection method			
PCR	6	4.97 (2.93–8.44)	49.2% (0.08)
FC	2	4.77 (3.31–6.87)	0 (0.70)
Adjustment			
Adjusted	4	4.56 (2.64–7.88)	48.9% (0.12)
Crude	4	5.18 (2.94–9.11)	24.4% (0.27)

### OS

**Fig. 5:**
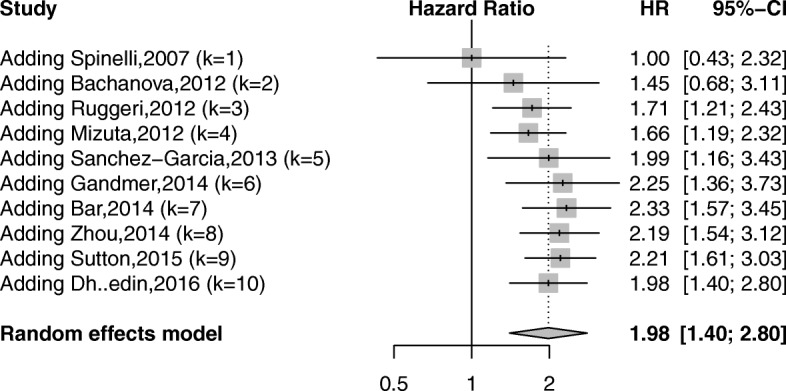
Forest plot showing the association between pre-transplant MRD and overall survival after allo-SCT

Ten studies showed data on OS outcome [19, 28, 29, 32, 35, 37–40, 42]. The adjusted HRs were directly obtained from four studies [19, 35, 37, 42] and indirectly derived from KM curves in six studies [28, 29, 32, 38–40]. The pooled data demonstrated that patients with ALL having positive MRD prior to allo-SCT had a significantly unfavorable OS (HR = 1.98; 95% CI 1.40–2.80; *P* < 0.05) (Fig. 5). A significant heterogeneity was revealed (*I*^2^ = 67.2%; *P* < 0.01).

In stratified analyses, the pooled data for the subgroups of adult patients and Asian population showed no statistical significance (Table 5). The heterogeneity was low in the three subgroups of the population and the subgroups of American or Asian studies. In a subgroup analysis, the exclusion of any specific study did not alter the pooled conclusion significantly.

**Table 5:** Subgroup analyses for the outcome of overall survival

Subgroups	No. of studies	HR (95% CI)	*I*^2^ (*P* value)
Population			
Adult	4	1.15 (0.86–1.53)	0 (0.73)
Children	3	2.40 (1.49–3.89)	37.7% (0.20)
Mixed adult and children	3	3.06 (1.98–4.70)	32.1% (0.23)
Design			
Prospective	6	1.68 (1.02–2.77)	60.1% (0.03)
Retrospective	4	2.37 (1.55–3.63)	64.8% (0.04)
Region			
Europe	5	2.05 (1.10–3.80)	83.0% (< 0.01)
USA	3	2.10 (1.50–2.94)	1.8% (0.36)
Asia	2	1.89 (0.99–3.62)	0 (0.32)
Detection method			
PCR	6	1.65 (1.07–2.55)	61.5% (0.02)
FC	4	2.53 (1.58–4.04)	58.1% (0.07)
Adjustment			
Adjusted	4	2.58 (1.27–5.26)	71.1% (0.02)
Crude	6	1.70 (1.18–2.44)	58.7% (0.03)

### NRM

**Fig. 6:**
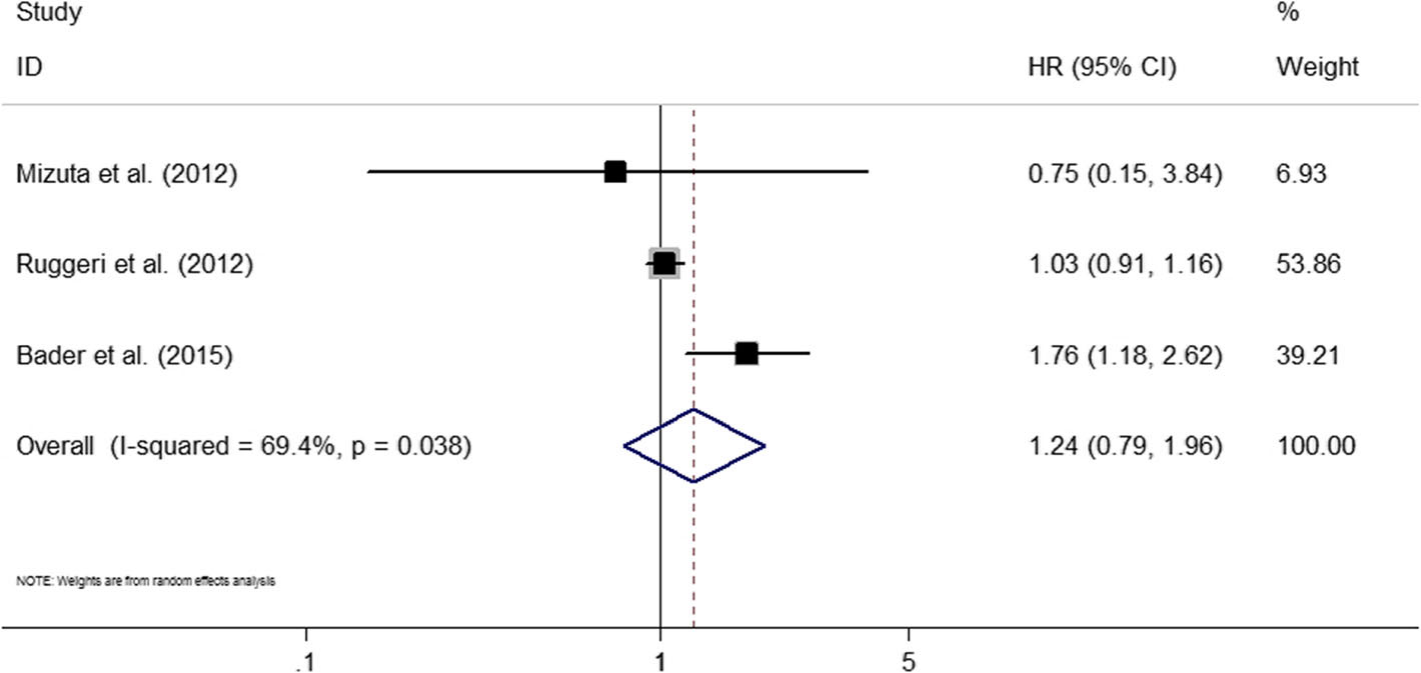
Forest plot showing the association between pre-transplant MRD and non-relapse mortality after allo-SCT

Three studies were eligible [19, 25, 40]. Positive MRD prior to transplantation was not associated with a higher rate of NRM (HR = 1.24; 95% CI 0.79–1.96; *P* = 0.35) (Fig. 6). A high heterogeneity was revealed (*I*^2^ = 69.4%; *P* < 0.05). Subgroup analyses or publication test was not conducted due to a limited number of studies. The three studies were excluded one by one, and the conclusion did not change significantly.

### Publication bias

**Fig. 7:**
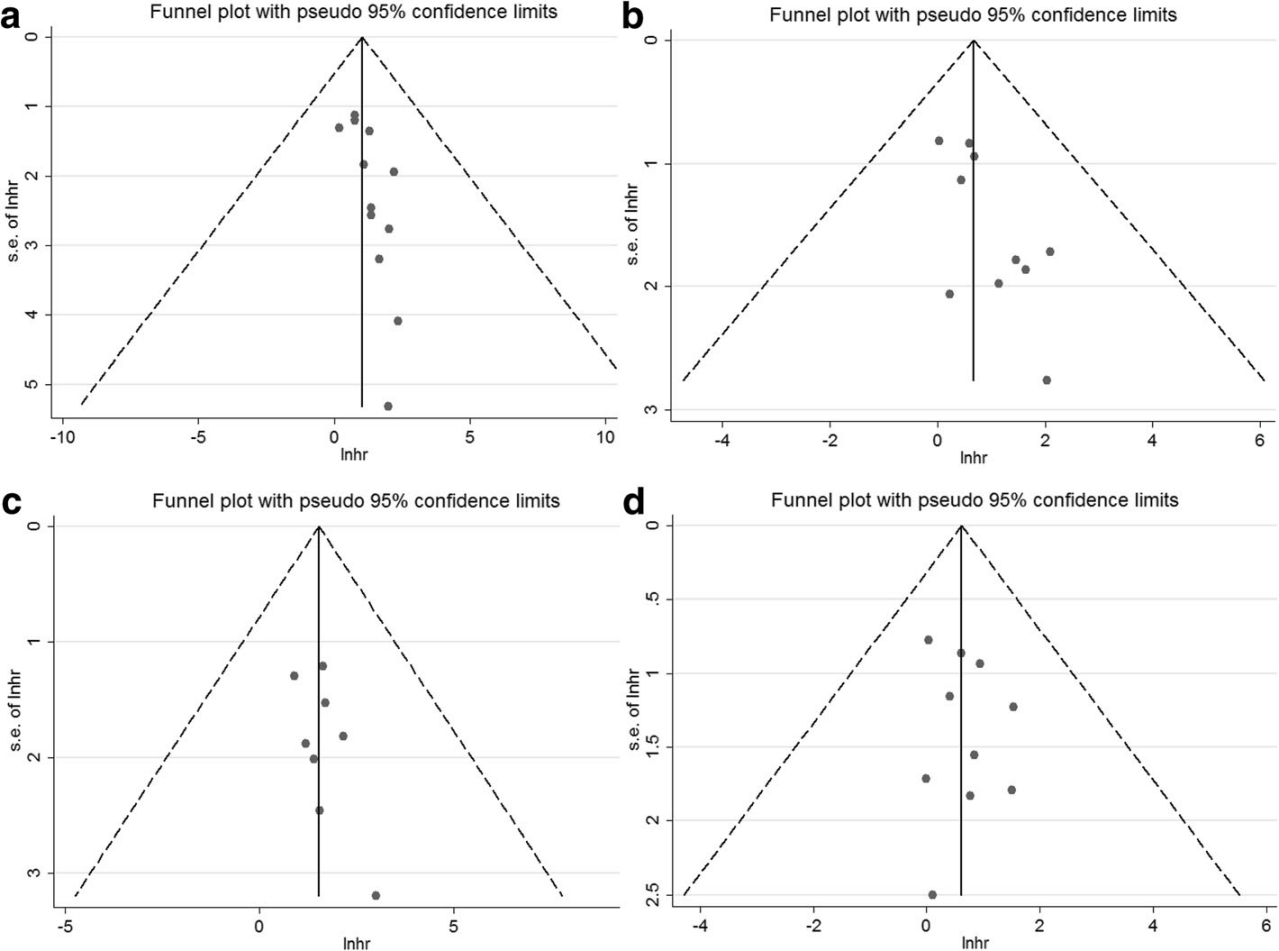
Funnel plots for the outcomes of relapse (**a**), relapse-free survival (**b**), event-free survival (**c**), and overall survival (**d**)

The publication bias for the outcomes of relapse, RFS, EFS, and OS was assessed. Notably, the funnel plots were asymmetrical for the outcome of relapse (Fig. 7a) and RFS (Fig. 7b). In contrast, the plots were symmetrical for the outcomes of EFS (Fig. 7c), and OS (Fig. 7d). When statistically assessed using the Egger’s test, the publication bias was statistically significant for relapse (*P* = 0.02) and RFS (*P* = 0.02), but not for EFS (*P* = 0.20) or OS (*P* = 0.49). Further, the results of Begg’s tests were examined, showing no publication bias for relapse (*P* = 0.06), RFS (*P* = 0.21), EFS (*P* = 0.39), and OS (*P* = 0.86).

## Discussion

The prognostic value of pre-transplant MRD was demonstrated in several ways. First, the collected data revealed that patients with positive MRD before allo-SCT had a higher cumulative incidence of relapse in the follow-up. In accordance, the RFS was much shorter for the MRD (+) patients. The EFS was a composited outcome defined as the time from allo-SCT to the first occurrence of relapse or death. Positive MRD before allo-SCT was also an independent negative predictor of EFS. Furthermore, patients with positive pre-transplant MRD were proved to have a significant dismal OS. To the best of my knowledge, this meta-analysis was the first to appraise the role of MRD assessment in the pre-transplant setting in patients with ALL.

The prognostic power of pre-transplant MRD, as well as the sources of heterogeneity, was examined by subgroup analyses. A careful statistical process was conducted with caution that several clinical covariates might interact with the role of MRD and potentially cause bias. Thus, the adjusted estimates were preferred. The most relevant studies paid attention to the well-known confounding factors of disease status (CR1 or CR2), age, sex, and genetic mutations. Notably, the pooled HRs from the adjusted multivariate analysis or crude analysis from KM curves uniformly demonstrated a significant association between pre-transplant MRD and the outcomes of relapse, EFS, and OS. Only for RFS, the crude analysis by three studies failed to show the significant correlation. The included studies using PCR assay mostly amplified the rearranged immunoglobulin and TCR gene segment in the leukemic clone. Only few studies tested the fusion gene of BCR-ABL1. The FC-based assay, by examining the immunophenotypes, was advantageous in terms of rapid process and readily available results. Subgroup analyses demonstrated that the statistical significance for relapse, RFS, EFS, and OS was estimated using either PCR or FC method. The leukemogenic events were different for pediatric and adult patients with ALL [43]. A higher prevalence of unfavorable genetic subtypes, such as the BCR-ABL1 fusion protein, was observed among older patients [2]. The latest National Comprehensive Cancer Network guideline stated that the OS decreased substantially with increased age for patients with ALL [1]. Interestingly, MRD was not found to be a significant predictor of dismal OS for adult patients with ALL. However, MRD prior to transplantation was a constant negative predictor among the outcomes of relapse, RFS, and EFS, regardless of discrepancies in patients’ ages. Compared with prospective studies, retrospective studies relied on data recall or information from previous medical records. However, a significant difference was not observed by analyzing retrospective or prospective studies alone.

Of note, when assessing the outcome of NRM, the MRD status prior to transplantation did not have a significant role. Myeloablative conditioning for MRD-positive patients versus reduced intensity conditioning for patients with undetected MRD might affect the NRM outcomes. A small number of studies might limit the statistical power. Additionally, some other factors might outweigh MRD in predicting NRM. Previous evidence suggested that younger adults had reduced post-transplant mortality. Myeloablation might be not feasible in patients older than 35 years because a higher toxicity was more commonly seen in these recipients [26, 44]. Thus, MRD might only be a subordinate factor for this outcome.

This meta-analysis had several strengths. It included 21 studies with a total of 2323 patients around the world. Comprehensive prognostic outcomes, including relapse, RFS, EFS, OS, and NRM, were evaluated. The prognostic value of MRD was appraised according to different assaying modalities, populations, and study designs. Both adjusted and crude data were presented. Largely, the association between MRD and outcomes remained stable among subgroups. It was confirmed that the detection of MRD was of considerable importance in identifying patients with poor outcome after allo-SCT. MRD was advocated to be a useful molecular biomarker for accurate triage of patients’ =pre-transplantation and preemptive escalation of post-transplant interventions [15].

This study also had several shortcomings. Several studies collected data retrospectively. Some studies had small sample sizes, which might have reduced the statistical power. Patients’ disease status of NRM might have biased this relationship. The inclusion criteria were heterogeneous, and patients were treated using varied chemotherapy protocols. The timing and duration of follow-up were inconsistent. Also, the definitions of assay-specific thresholds and the lack of one universal detection method or testing target were heterogeneous among the included studies. No consensus was reached regarding the standardization of MRD measurement. Furthermore, it failed to give strong justification for providing a quantitative assessment of the influence of pre-transplant MRD. A multitude of confounding factors, such as the use of tyrosine kinase inhibitors, the pre-transplant remission type (CR1 or CR2), donor source, and graft-versus-leukemia, were not sufficiently adjusted in many studies when analyzing the impact of MRD. In fact, these factors were even inconsistent within an individual study [25, 40]. Even for studies that reported adjusted HRs, the degree of adjustment largely varied. The subgroup findings should be considered as exploratory, and thus would need to be tested in original studies. Finally, this study was conducted with summary statistics rather than with individual data, which might have ignored the impact of some covariates on the outcomes at the patient level. The availability of data from individual patients could resolve this problem and increase the power of meta-analysis.

Future studies should aim to decide how best to use the prognostic information of MRD. Several ways can be considered to improve the outcomes for MRD (+) patients at transplantation. Pre-transplantation treatment with non-cross-resistant agents might be helpful in decreasing the residual malignant clone [14, 40]. Preemptive immunotherapy or chemotherapy might be beneficial during the post-transplantation stage [45, 46]. Lankester et al. preliminarily revealed that alloimmune intervention after allo-SCT was feasible in reducing residual leukemic cells [17]. Further, a randomized trial should be performed on patients with ALL in complete remission who had positive MRD and received either allo-SCT or additional novel chemotherapy before transplantation.

## Conclusions

In conclusion, this meta-analysis provided evidence that positive MRD prior to allo-SCT was associated with higher relapse and poor survival in patients with ALL. Allo-SCT appeared to be insufficient for some patients with positive MRD at transplantation. The findings of this study suggested the rationale for future studies to prevent relapse and improve survival for this group of high-risk patients.

## Additional file

Additional file 1: **Table S1.** Quality assessment of included studies using the Newcastle–Ottawa Scale (maximum score of 9). (DOCX 19 kb).

## Abbreviations

ALL: Acute lymphoblastic leukemia
allo-SCT: allogeneic Stem cell transplantation
BCR-ABL: Breakpoint cluster Region-Abelson
DFS: Disease-free survival
EFS: Event-free survival
FC: Flow cytometry
HRs: Hazard ratios
KM: Kaplan–Meier
LFS: Leukemia-free survival
MRD: Minimal residual disease
NRM: Nonrelapse mortality
OS: Overall survival
PCR: Polymerase chain reaction
RFS: Relapse-free survival
TCR: T-cell receptor
