# Supplementary material for: Influence of pre-transplant minimal residual disease on prognosis after Allo-SCT for patients with acute lymphoblastic leukemia: systematic review and meta-analysis
Source: BMC Cancer. 2018 Jul 23;18:755. doi: 10.1186/s12885-018-4670-5 (PMC6056932; doi:10.1186/s12885-018-4670-5)
Supplement: Supplementary file 1 — Table S1. Quality assessment of included studies using the Newcastle–Ottawa Scale (maximum score of 9). (DOCX 19 kb). [file 12885_2018_4670_MOESM1_ESM.docx]

Additional file 1: **Table S1** Quality assessment of included studies by the Newcastle–Ottawa Scale (maximum score of 9).

|  | Selection | | | | Comparability | Outcome | | |  |
| --- | --- | --- | --- | --- | --- | --- | --- | --- | --- |
| Author (year) | Representativeness  of the MRD (+)  patients | Selection of the MRD (–)  patients | Ascertainment  of pre-transplant MRD | Outcomes were not present at start of study | Comparability on the basis of the design or analysis | Assessment  of outcome | Adequate follow-up duration  (≥ 5 years) | Adequate  follow-up rate  (>80%) | Overall  quality |
| Knechtli et al. (1998) | 1 | 1 | 1 | 1 | 2 | 1 | 0 | 1 | 8 |
| Bader et al. (2002) | 1 | 1 | 1 | 1 | 0 | 1 | 1 | 1 | 7 |
| Sramkova et al. (2007) | 1 | 1 | 1 | 1 | 0 | 1 | 0 | 1 | 6 |
| Spinelli et al. (2007) | 0 | 1 | 1 | 1 | 0 | 1 | 0 | 1 | 5 |
| Bader et al. (2009) | 0 | 1 | 1 | 1 | 2 | 1 | 0 | 1 | 7 |
| Elorza et al. (2010) | 1 | 1 | 1 | 1 | 0 | 1 | 0 | 1 | 6 |
| Lankester et al. (2010) | 0 | 1 | 1 | 1 | 0 | 1 | 1 | 1 | 6 |
| Doney et al. (2011) | 1 | 1 | 1 | 1 | 2 | 1 | 1 | 1 | 9 |
| Bachanova et al (2012) | 1 | 1 | 1 | 1 | 2 | 1 | 0 | 1 | 8 |
| Ruggeri et al. (2012) | 1 | 1 | 1 | 1 | 2 | 1 | 0 | 1 | 8 |
| Mizuta et al. (2012) | 0 | 1 | 1 | 1 | 2 | 1 | 0 | 1 | 7 |
| Sanchez-Garcia et al. (2013) | 1 | 1 | 1 | 1 | 2 | 1 | 1 | 1 | 9 |
| Balduzzi et al. (2014) | 1 | 1 | 1 | 1 | 2 | 1 | 1 | 1 | 9 |
| Gandemer et al. (2014) | 1 | 1 | 1 | 1 | 2 | 1 | 1 | 1 | 9 |
| Tucunduva et al. (2014) | 0 | 1 | 1 | 1 | 2 | 1 | 0 | 1 | 7 |
| Logan et al. (2014) | 1 | 1 | 1 | 1 | 0 | 1 | 0 | 1 | 6 |
| Zhou et al. (2014) | 1 | 1 | 1 | 1 | 2 | 1 | 0 | 1 | 8 |
| Bar et al. (2014) | 1 | 1 | 1 | 1 | 0 | 1 | 0 | 1 | 6 |
| Bader et al. (2015) | 0 | 1 | 1 | 1 | 2 | 1 | 0 | 1 | 7 |
| Sutton et al. (2015) | 1 | 1 | 1 | 1 | 2 | 1 | 1 | 1 | 9 |
| Dh’edin et al. (2016) | 0 | 1 | 1 | 1 | 0 | 1 | 0 | 1 | 5 |
